# An integer programming model to assign patients based on mental health impact for tele-psychotherapy intervention during the Covid–19 emergency

**DOI:** 10.1007/s10729-020-09543-z

**Published:** 2021-04-11

**Authors:** Andrés Miniguano-Trujillo, Fernanda Salazar, Ramiro Torres, Patricio Arias, Koraima Sotomayor

**Affiliations:** 1 Maxwell Institute for Mathematical Sciences, The University of Edinburgh, Bayes Centre, 47 Potterrow Edinburgh, United Kingdom; 2 Department of Mathematics – Escuela Politécnica Nacional, Quito, Ecuador; 3 Ecuadorian Association of Evidence-Based Psychology and Psychotherapy (AEPPBE), Quito, Ecuador; 4 Human Cognitive Neuroscience, Psychology, The University of Edinburgh, Edinburgh, United Kingdom

**Keywords:** Integer programming, Logistic regression, WLSMV, AAQ-II, Tele-psychotherapy, SARS–CoV2

## Abstract

The Covid–19 pandemic challenges healthcare systems worldwide while severely impacting mental health. As a result, the rising demand for psychological assistance during crisis times requires early and effective intervention. This contributes to the well-being of the public and front-line workers and prevents mental health disorders. Many countries are offering diverse and accessible services of tele-psychological intervention; Ecuador is not the exception. The present study combines statistical analyses and discrete optimization techniques to solve the problem of assigning patients to therapists for crisis intervention with a single tele-psychotherapy session. The statistical analyses showed that professionals and healthcare workers in contact with Covid–19 patients or with a confirmed diagnosis had a significant relationship with suicide risk, sadness, experiential avoidance, and perception of severity. Moreover, some Covid–19-related variables were found to be predictors of sadness and suicide risk as unveiled via path analysis. This allowed categorizing patients according to their screening and grouping therapists according to their qualifications. With this stratification, a multi-periodic optimization model and a heuristic are proposed to find an adequate assignment of patients to therapists over time. The integer programming model was validated with real-world data, and its results were applied in a volunteer program in Ecuador.

## Highlights

- Evidence of psychological risk was found in the Ecuadorian population
- Working with Covid–19 patients has a significant relationship with psychological risk
- Multi-periodic optimization models the assignment of patients to therapists
- A heuristic efficiently finds an assignment of patients to therapists
- Demonstrates a successful real-world application of integer programming during the Covid–19 emergency

## Introduction

New restrictions and sudden life changes with profound effects not only in physical health but also in mental health reflect the complex impact of the SARS–CoV2 (Covid–19) pandemic in economic, social, and political systems [67]. WHO (World Health Organization) encouraged governments and individuals to keep up with for physical and psychological care [84]. Nevertheless, worldwide, the pandemic crisis has caused mental health problems related to anxiety, depression, and increased suicide risk [21, 27]. These affect not only the general population but also front-line workers like doctors, nurses, and psychologists.

A country facing these pandemic-related challenges is Ecuador. Guayaquil, the second-largest city, was affected to no small extent reaching, at the time, the highest per capita Covid–19 death toll in Latin America and the Caribbean [48]. On 16th March, the Ecuadorian president declared a state of emergency, and over the following days, a country-wide lock-down was set in place. Until mid-May, the number of confirmed cases was around 35 000, sanitary systems continued their labor while reaching their maximum capacity, and only essential workers were allowed to leave their homes. Regardless, the government eased its lock-down rules since the end of May. The latest official epidemiological report of the Ministry of Public Health (MSP) is from 3rd May and reports 20 937 confirmed cases and 17 535 possible cases of Covid–19 [54]. The figures have grown ever since, as, according to MSP, there were more than 50 000 confirmed cases of Covid–19 in the country until mid-June, and Guayaquil continued leading the statistics of positive diagnoses [52].

To face the difficulties associated with Covid–19, the Ecuadorian government implemented an emergency phone line (171), and the MSP launched guidelines to address mental health needs within the public [40]. The latter measure goes in the same lines as [24], who described how psychological counseling open-telephone helplines quickly became important mechanisms in addressing psychological issues during SARS and natural disasters in China. Also, the authors encouraged continuing the improvement of psychological intervention during crisis events.

Following Governmental’s response, many Non-Governmental Organizations (NGOs) joined efforts with public institutions to provide accessible mental health services. As a result, the role of NGOs in psychological practices has been crucial since early intervention has proved effective during crisis events [76, 85]. There are many NGOs in Ecuador that provided free services due to the Covid–19 emergency: One of them was the Ecuadorian Association of Evidence-Based Psychology and Psychotherapy (AEPPBE) [*Asociación Ecuatoriana de Psicología y Psicoterapia Basada en Evidencia*]. Since, Tele-behavioral Health is an effective way to approach a wide range of psychological problems [43, 83], AEPPBE organized a free volunteer work regarding the guidelines of the APA (American Psychology Association) [39, 49] and the Ecuadorian guidelines on Telepsychology of MSP [53].

AEPPBE launched a volunteering program consisting of two consecutive stages: (a) an online psychological assessment and (b) a brief evidence-based psychotherapy intervention. The former focused on measuring several trans-diagnostic variables from contextual therapies and crisis intervention. The objective of this assessment was to quantify the impact of the crisis on the population’s mental health. For this, the group of variables included experiential avoidance [35], perception of severity [3, 23], and suicide risk. Additionally, the assessment also considered socio-demographic information such as age, profession, if participants were under quarantine, if they have been diagnosed with Covid–19, and if they had been in contact with someone diagnosed with Covid–19.

Post-traumatic stress disorder, depression, anxiety, substance use or abuse, or increasing suicide rates are some psychological outcomes of crisis events [29, 67]. Sanderson et al. [73] described in detail several variables impacting mental health during a pandemic, opening a call for evidence-based cognitive-behavioral strategies to reduce the emotional suffering associated with the Covid–19 crisis. In addition, experiential avoidance is a trans-diagnostic variable in anxiety and depression [44]. Recent research showed experiential avoidance as a predictor of anxiety, phobias, and trauma [5]. Thus, research has shown that interventions exploring and reducing experiential avoidance are beneficial for ameliorating various psychological difficulties [12].

Perceived severity refers to an individual’s perception of the seriousness of contracting an illness or disease. Prior research showed a link between this variable on Covid–19 and mental health problems. It has been explored in combination with other mediator factors in front-line healthcare professionals of Pakistan to establish priority psychological aid [71]. Moreover, Li et al. [45] found a negative correlation between Perceived Severity of Covid–19 and mental health problems. Besides, the authors mentioned another research with 4 607 Chinese people showing how the perceived severity of Covid–19 is linked to several undesirable outcomes (e.g., sleep problems or negative emotions).

Furthermore, besides the disease, suicide is a major source of concern during this pandemic. Accompanying all the stigma surrounding suicide, its conjunction with Covid–19 isolation and over-saturated emergency telephone lines, Gunnell et al. [32] proposed that rates might increase. Thakur et al. [77] explained how suicide stigma, basic psychology, the failure of individuals to act, and the lack of government involvement are linked to the incidence of suicide during the Covid–19 pandemic. Consequently, addressing suicide risk is one of the critical preventive strategies [68, 75]. Throughout this period of isolation, diverse contextual factors can influence and anticipate suicide rates in Ecuador.

Evidence from the SARS epidemic and the Influenza A-H1N1 pandemic states the relevance of early intervention in mental health across different disease-affected populations [42, 50, 64]. During pandemics, healthcare staff is exposed to contagious materials, high levels of stress, sleep deprivation, or compassion fatigue [14, 58, 65]. Thus, health workers have a higher probability and disposition to develop mental health anguish and disorders [69]. An important component to effectively alleviate pandemic-related distress is to prioritize psychological intervention for healthcare professionals [25, 63]. Inadequate interventions to help them would lead to quitting and future psychological problems [32, 56].

Strategic alliances during the pandemic will lead to effective contextual intervention. As the odds of significant consequences in mental health are high [21, 72], guidelines from the Inter-American Psychological Society state that *“the presence of psychologists in the mass media is crucial to carry a message of calm to the population”* [28]. Moreover, Zaka et al. [88] highlighted the urgent need to implement mental health interventions to front-line medical staff working with Covid–19. Ordered and brief intervention with healthcare staff seems to be effective [13]. International efforts demonstrated the effectiveness of Tele-psychology response, requiring a well-organized system to effectively address the population’s needs and prevent future psychological negative impacts [46, 81]. In Ecuador, the same approach was adopted without control, and high stress levels were reported in volunteers. Known barriers imposed by technology, accessibility, or limited clinic capacity [62] combined with exiguous governmental emergency lines responses and assignments performed without clinical guidance. Thus, the need for efficacious intervention to provide mental health strategies to volunteers and the public became imperative.

As a result of the preceding discussion, AEPPBE provided help to the public since the start of the sanitary emergency in Ecuador. Sixty-three volunteers, conformed by psychologists, psychotherapists, and final-year students of psychological sciences, received online training to provide tele-psychological assistance. The intervention was based on a protocol of a unique session addressing main issues from a contextual approach. Nevertheless, the wide range of patients and their symptoms increased the difficulty of finding a proper assignment. The presence of Covid–19 contact or suicide risk could not be assigned directly to any therapist, due to their academic background, capabilities, or training. However, since the volunteer work of AEPPBE was organized with scarce resources and aiming to face the crisis quickly, assignments were made by two members of AEPPBE. The process required to manually match known therapists’ information with the needs of each patient; thus, it pressed for additional work overload and reported incorrect assignments before the development of this work. To adequately address this problem, an Integer Linear Programming model can be formulated.

Linear and Integer Programming are techniques for solving constrained optimization problems and a valuable tool for decision making. Specifically, in the case of healthcare, several studies have been reported in the literature. Vieira et al. [80] proposed a stochastic mixed-integer linear programming model. It allocates radiation therapy technologists to multiple operations in radiotherapy over a set of scenarios of patient inflow. The model aimed to maximize the expected number of patients completing pre-treatment within the waiting time target. In a medical records department, Chan et al. [20] described quantitative models to determine the optimal worker schedule. There, a simulation model was designed to represent the workflow’s transcription function. In [22], the authors presented a mixed-integer programming model for allocating traumatic brain injury treatment units to those patients currently in the Department of Veterans Affairs. The model minimized treatment costs, patient lodging, travel costs, and penalty costs of foregone treatment revenue and excess capacity utilization. Yang et al. [86] proposed a simulation-based optimization method for ambulance allocation, with a simulated model emulating the operational processes and evaluate the performance of an ambulance allocation plan in an uncertain environment. Zhang et al. [89] presented a two-stage bi-objective mixed-integer model to locate and allocate mobile blood collecting facilities; this process directly impacts the timely supply of blood. At last, Yu et al. [87] proposed to optimize the resource management and allocation scheme using Timed Colored Petri net, dynamic simulation, and continuous optimization. To the best of our knowledge, the literature review did not reveal studies on linear programming applications with a compartmental structure for telepsychology.

Lacking enough skilled personnel can negatively impact patient outcomes, including mortality [2]. A particular well-studied case is on nurse scheduling to deal with shortages at hospitals, then Operations Research techniques are useful to provide efficient schedules. One of the first optimization models in this setting was introduced by Warner and Prawda [82], where nursing personnel scheduling is formulated as a mixed-integer quadratic programming model. More recently, Bellanti et al. [10] proposed a local search approach based on partial solutions to be completed with a greedy procedure, and these solutions were applied at an Italian hospital. Bard and Purnomo [7] introduced an integer programming model considering individual preferences when constructing a monthly nurse schedule. The model was solved using a column generation technique, showing that it can cope with nurse scheduling processes. Later, Bester et al. [11] constructed a nurse schedule with a tabu search approach for a large psychiatric hospital in South Africa. Using exact optimization methods, Beliën and Demeulemeester [9] proposed an integrated model for nurses and operating room staff. Schoenfelder et al. [74] presented a multi-stage stochastic programming model for nurse scheduling and quick-response decisions. It focused on scheduling a mix of unit and cross-trained float nurses with the additional option of transferring patients between units under uncertain patient demand. Often, a combination of scheduling with vehicle routing problems is also studied. Fikar and Hirsch [26] proposed a model for health-care operations, where nurses are scheduled and routed for home health-care. Detailed surveys are found in [17] and [34].

The problem of assigning patients for medical treatment slots is often also considered in medical scheduling. As timely access to appointment assignation is important for achieving better medical outcomes and patient satisfaction, healthcare networks have recently grown larger by including appointment slot allocations, which cannot be managed manually. Griffiths et al. [30] sought to compute a timetable for physiotherapy treatment formulating the problem as a multi-objective combinatorial optimization problem. Here, a three-stage local search approach was used to provide an approximate solution. Pérez et al. [61] solved the problem for assigning patient treatment slots and resources in nuclear medicine using a stochastic online scheduling algorithm. Kuiper and Mandjes [40] used the transient distribution of the tandem queue to set up schedules and compared online and offline approaches with quadratic and linear loss functions. The problem of assigning the physician most qualified to treat a particular patient can be found in [79], where a model of dynamic advance scheduling with two patient classes is proposed alongside an efficient algorithm to compute the optimal policy for assigning patients to exam days. Parizi and Ghate [59] considered assignment problems where different types of appointment requests arrive dynamically and stochastically. The authors proposed a Markov-decision process model together with an approximate dynamic programming solution algorithm while considering no-shows, cancelations, and overbooking. An extensive review on assignment for outpatient services can be found in [19] and [1].

Since there is no local example of an effective psychological response addressing Covid–19 in volunteering through online assessment, two goals are pursued in this work. The first is to discover relations between the psychological variables explored in the online assessment of AEPPBE volunteer work. Then, patients and therapists are stratified to define and treat prioritized groups, namely healthcare workers, Covid–19 patients, and suicide patients. The second goal is to properly develop this assignment using Integer Programming, where therapists provide adequate interventions in a unique session based on their competences, preferences, and capabilities. Formally, given a set of patients categorized according to psychological criteria and therapists grouped by their aptitudes to treat each patient’s category, the model seeks an optimal assignment between the two by maximizing their affinity. This must satisfy (a) the compatibility between categories, (b) the maximum number of patients allowed per therapist, and (c) a homogeneous workload distribution among therapists and groups. The assignment must be performed periodically.

## Methodology

In order to meet the goals of this study, a two-phase methodology is presented. The first phase analyzes the relationship among the psychological criteria proposed in the online assessment to identify prioritizing treatment categories. A correlational and predictive study was chosen with moderation and cross-sectional cohort analysis to meet the stated objective. The second phase uses the former results to provide an adequate assignment of patients to volunteer therapists over time. A multi-periodic optimization model is proposed together with a heuristical solution method to solve this problem. These Integer Programming techniques are later applied and validated with instances from real-world data.

In advance, ethical considerations were addressed, providing informed consent for the use and handling of the data. The data was collected in the framework of the voluntary social support initiate from the AEPPBE in response to the psychological outcomes from Covid–19 lockdown in the population. Thus, the data is from a cross-sectional non-probabilistic accidental sample. Data was collected with Google Forms, surveying the people who expressed their willingness to fill out the form. People who fulfilled the form were contacted to concrete an appointment for a unique psychotherapy sessions regarding psychological impacts of the Covid–19 pandemic. Prior to the analysis, raw data were converted to numerical scores. These were used to analyze the psychological criteria used to evaluate patients and therapists information.

### Participants

#### Patients

The collected sample was made up of 525 people from different regions of Ecuador with an average age of 34 (*SD* = 12.87) years. Of these, 71.2*%* are women, and 28.8*%* are men. These percentages align with [47] findings where men are less inclined than women to seek help for mental health issues. In addition, 10*%* are health professionals, and 9*%* have been in contact with patients diagnosed with Covid–19 in an occupational context.

#### Volunteer Therapists

The volunteer sample consisted of 63 volunteers, from which 42 were psychologists, and the 21 left were Psychology students in the final year of their studies. Therapists filled a questionnaire collecting the maximum number of patients they wanted to treat, their line of intervention, their competences, and their preferences. Within the practitioners, there was a mixture of psychological approaches. Most of them based their practice on Cognitive Behavioural Approaches 59*%* (*n* = 36), followed by Contextual Approaches 50.8*%* (*n* = 31), and Systemic Family Psychotherapy Approaches 23*%* (*n* = 14). From the total number of therapists, 33 had some training in suicide intervention, but only 63.3*%* of them wanted to provide voluntary assistance to patients with Suicide Risk.

### Instruments

#### Acceptance and Action Questionnaire - II

(AAQ-II; Bond et al. [15]. Spanish adaptation of Patrón Espinosa [60] in the Mexican population. This scale is a generic measure of experiential avoidance (EA) and psychological acceptance that has better internal consistency (*α* = 0.89) than the Spanish adaptation of the AAQ-I of Barraca Mairal [8] (*α* = 0.74), and it has the same level of consistency as the adaptation to the Spanish population of the AAQ-II from Ruiz et al. [70] (*α* = 0.89). It contains ten items, such as “I can remember something unpleasant without causing me discomfort,” which are graded on a seven-point Likert scale, indicating the highest scores, the highest degree of EA, and the lowest in experiential acceptance.

#### Perception of Severity.

The Subjective Severity Scale of Alzugaray et al. [3] was used. This scale measures individuals’ perception of a specific stressful event; in this case, the health emergency given by the Covid–19 pandemic. Three items form the scale (e.g., *To what degree do you feel that your life was altered as a result of the coronavirus crisis?)*, and it is evaluated on a Likert scale from 0 (“not altered”) to 4 (“severely altered”).

#### Brief scale of triage in emergencies

This scale briefly measures a variety of psychological symptoms related to emergencies. Two questions were used, one to measure sadness and the desire to harm or kill oneself (suicide risk). The scale obtains a dichotomous value response (yes/no). This instrument was used to evaluate the risk of participants briefly.

#### Socio-demographic Questionnaire - Participant Form

A short survey was prepared. It collected personal data such as name, age, sex, educational level, profession, telephone number, if the participant works with Covid–19 patients, if the participant had contact with Covid–19 patients, and if they have been diagnosed with Covid–19.

#### Capabilities and Preferences Questionnaire - Therapist Form

This was a short questionnaire where therapists provided some identifying information such as their name, ID, educational level, preferred psychotherapy approach, and training in suicide intervention. Additionally, they stated their preference related to the maximum number of patients they could treat per week and their competence to treat different types of patients.

## First phase: correlation analysis to compute categories and groups

### Data analysis

Due to the nature of the variables, correlations were evaluated using Pearson’s *χ*^2^ test-statistic. Two logistic regressions were performed to establish the influence of the predictor variables on the outcome variables: one on the Perception of Severity and another on the single item evaluating Sadness. Then, a Path Analysis was performed to evaluate the level of influence of the studied variables, the robust Weighted Least Square Mean and Variance adjusted method (WLSMV) with polychoric matrices was used as an estimation method, as it does not assume normally distributed variables and is a suggested option for modelling categorical data [6, 16]. The *χ*^2^ statistic was used as an adjustment index, however, due to its sensitivity to the sample size, the Comparative Fix Index (CFI), the Tucker-Lewis Index (TLI), and the Root Mean Square Error of Approximation (RMSEA) were also used with their confidence intervals. Here, a goodness of fit is considered when the *χ*^2^ statistic has a *p* value greater than 0.05, the CFI and the TLI reach values greater than 0.95, and the RMSEA is less than 0.08, with a confidence interval width not exceeding 0.10. For all these analyzes, the statistical software SPSS Statistics 23 [36] and MPLUS 7.0 [55] were used.

### Results

The correlations among the studied variables are presented in Table 1. It is possible to verify that Profession showed a significant correlation with the Subjective Perception of Severity, Suicide Risk, and Experiential Avoidance. Moreover, Working with Covid–19, Quarantine, and Diagnosis of Covid–19 showed a relation only with the Perception of the Severity of the emergency. Thus, these results show how different variables should be consider in the classification of patients. A multi-variable approach to prioritize patients should lead to an effective treatment.

**Table 1:** Test of *χ*^2^

Criteria	Perception of	Suicidal	Experiential
	Severity	Risk	Avoidance
	(*χ*^2^,*p*)	(*χ*^2^,*p*)	(*χ*^2^,*p*)
Profession	(15.58,.000)	(4.36, 0.37)	(8.26,.004)
Working with	(20.77,.000)	–	–
Covid–19			
Quarantine	(20.77,.000)	–	–
Covid–19 diagnosis	(7.66, 0.22)	–	–

Table 2 shows the results of the logistic regressions on the Subjective Perception of Severity and Suicide Risk by using Working with Covid–19, Profession, and Covid–19 positive diagnosis. The analysis showed that Working with Covid–19 and Profession predict the Subjective Perception of the Severity of the health crisis. Table 3 holds the results of another logistic regression showing that Suicide Risk is predicted by Age, Profession, and Perception of the Severity of the crisis. It was found that being a health professional during quarantine predicts Suicide.

**Table 2:** Logistic regression analysis of Subjective Perception of Severity and Suicidal Risk with Covid–19 related variables

	Subjective Perception of Severity			Suicide Risk		
	*SD*	*B* value	*p* value	*SD*	*B* value	*p* value
(Constant)	.611	− 2.53	< .001	–	–	–
Working with	.378	1.34	< .001	.480	.127	.79
Covid–19						
Profession	.323	.91	.005	.627	− 1.26	.04
Covid–19 diagnosis	.454	.81	.077	–	–	–

**Table 3:** Logistic regression analysis of Suicidal Risk with demographic variables

	Suicide Risk		
	*SD*	*B* value	*p* value
(Constant)	.596	− 1.95	< .001
Age	.012	− .036	.002
Profession	.623	− 1.44	.021
Perception of Severity	.071	.174	.014

Subsequently, a directional analysis was carried out through a Path Analysis between the categorical variables via the estimation method WLSMV. The goodness of fit obtained by the model was good, where the correlated variables Profession, Working with Covid–19, Covid–19 Diagnosis, Perception of Severity, and Experiential Avoidance, predict Sadness and Suicide Risk. This model showed CFI = 0.95, TLI = 0.81, *χ*^2^/*df* = 8, RMSEA = 0.11, and a confidence interval of (0.09,0.15). In Fig. 3, the standardized factor weights are presented. These results denote a linear relation among Suicide Risk and variables as Age, Profession, and Perception of Severity, Working with Covid–19, and Covid–19 Diagnosis. Thus, prioritizing psychological intervention for patients with higher scores in these variables should be effective in the long run.

### Categorization and grouping

#### Patients

**Fig. 1:**
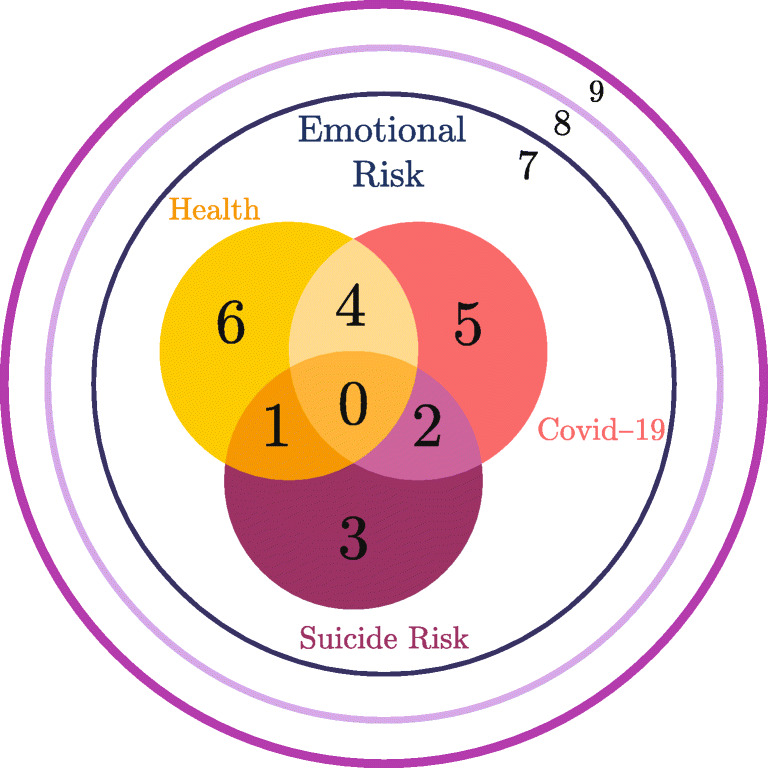
Patients Categories

After the statistical analysis, the patient sample was divided into categories, as pictured in Fig. 1. Here, 0 represents the category with the highest priority, and category 9 is the one with less treatment priority. The categories within the classification aim to prioritize patients related to healthcare services, Covid–19 patients, and the presence of high Suicide Risk. For instance, Category 0 describes patients with the most significant psychological risk associated with distress, suicide, and other mental health outcomes. Categories 1 to 9 describe upper levels where patients show fewer risk factors or less intensity of symptoms. Particularly, Category 9 includes patients with Low Emotional Risk, non-suicidal risk, non-existence diagnosis of Covid–19, and no contact with Covid–19 patients. This is showed in Table 4.

**Table 4:** Particularities of patient categories

Category	Covid–19 diagnosis	Suicide Risk	Covid–19 Contact	Health Worker	Experiential Avoidance		
					High	Medium	Low
0	$\checkmark $	$\checkmark $	$\checkmark $	$\checkmark $	$\checkmark $	$\checkmark $	$\checkmark $
1	–	$\checkmark $	$\checkmark $	$\checkmark $	$\checkmark $	$\checkmark $	$\checkmark $
2	$\checkmark $	$\checkmark $	$\checkmark $	–	$\checkmark $	$\checkmark $	$\checkmark $
3	–	$\checkmark $	–	–	$\checkmark $	$\checkmark $	$\checkmark $
4	$\checkmark $	–	$\checkmark $	$\checkmark $	$\checkmark $	$\checkmark $	$\checkmark $
5	$\checkmark $	–	–	–	$\checkmark $	$\checkmark $	$\checkmark $
6	–	–	$\checkmark $	$\checkmark $	$\checkmark $	$\checkmark $	$\checkmark $
7	–	–	–	–	$\checkmark $	–	–
8	–	–	–	–	–	$\checkmark $	–
9	–	–	–	–	–	–	$\checkmark $

#### Therapists

**Fig. 2:**
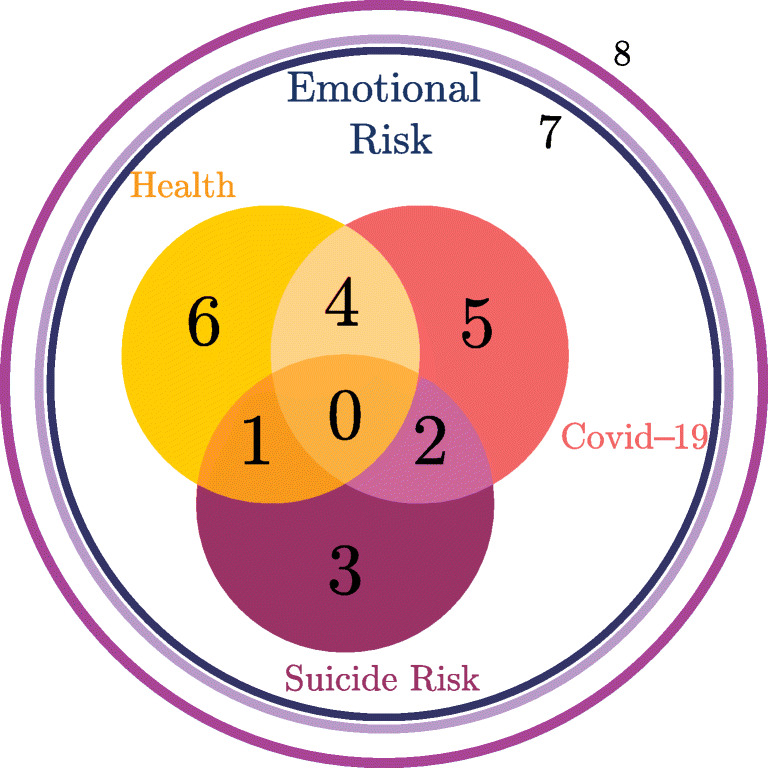
Therapist Categories

In order to address each category’s needs, the therapist sample was classified related to their results in the Capabilities and Preferences Questionnaire, see Fig. 2.

Table 5 describes the different Groups collecting each therapist. These Groups intersect with each other; thus, a Group 1 therapist can treat patients in Category 1, Category 6, and Category 3. Only therapists in Group 0 can treat any category of patients. The Capabilities and Preferences Questionnaire determined if a therapist had competence in suicide intervention and their preference related to patients. This preference assessed if they wanted to intervene with Covid–19 patients, health workers, or both of them. The combination of both, preferences and capabilities data, determines the qualification of a therapist to treat a category of patients or not. Lastly, volunteer Psychology students were automatically assigned to Group 8, regardless of their preferences.

**Table 5:** Particularities of therapist groups

Group	Covid–19 diagnosis	Suicide Risk	Covid–19 Contact	Health Worker	Experiential Avoidance		
					High	Medium	Low
0	$\checkmark $	$\checkmark $	$\checkmark $	$\checkmark $	$\checkmark $	$\checkmark $	$\checkmark $
1	–	$\checkmark $	$\checkmark $	$\checkmark $	$\checkmark $	$\checkmark $	$\checkmark $
2	$\checkmark $	$\checkmark $	$\checkmark $	–	$\checkmark $	$\checkmark $	$\checkmark $
3	–	$\checkmark $	–	–	$\checkmark $	$\checkmark $	$\checkmark $
4	$\checkmark $	–	$\checkmark $	$\checkmark $	$\checkmark $	$\checkmark $	$\checkmark $
5	$\checkmark $	–	–	–	$\checkmark $	$\checkmark $	$\checkmark $
6	–	–	$\checkmark $	$\checkmark $	$\checkmark $	$\checkmark $	$\checkmark $
7	–	–	–	–	$\checkmark $	$\checkmark $	$\checkmark $
8	–	–	–	–	–	–	$\checkmark $

## Second phase: optimal assignment of patients to therapists

In this section, an integer programming model is proposed for the assignment of patients to therapists based on the categorization and grouping developed in Section 3.3. Then two solution heuristics and computational results are presented. Table 10 from ?? summarizes the notation used throughout the section.

### Notation

The planning horizon is divided into *ρ* periods represented by a countable set *P* = {0,…,*ρ* − 1}. For each period *p* ∈ *P*, let *S*^*p*^ be the set representing the patients to be assigned, and *T*^*p*^ the set containing all the volunteer therapists. According to Section 3.3.1, the set of patients is always partitioned in *κ* categories, which do not depend on *P*. Let $\mathcal {C} = \{ 0, \ldots , \kappa -1 \}$ be the set of categories, then

$$ S^{p} = \underset{\ell \in \mathcal{C}}{\bigcup} {S}_{\ell}^{p}  \qquad\qquad \forall p\in P. $$

Further, a vector collecting the number of patients in every category at each period is defined as *b*^*p*^, where $ b_{\ell }^{p} $ is the cardinality of the set ${S}_{\ell }^{p}$, for each category $\ell \in \mathcal {C}$. Here, if there are no patients in category *ℓ* for period *p*, that is ${S}_{\ell }^{p}$ is an empty set, then ${b}_{\ell }^{p}$ is zero.

In a similar fashion, therapists are collected by their qualification in *σ* fixed groups independent of *P*. These are indexed by the set *Q* = {0,…,*σ* − 1} (see Section 3.3.2); thus,

$$ T^{p} = \bigcup_{\nu \in Q} T_{\nu}^{p}  \qquad\qquad \forall p\in P. $$

This qualification allows therapists to treat a set of categories of patients represented by the sets $F_{q_{t}} \subseteq \mathcal {C}$, where *q*_*t*_ ∈ *Q* is the rank of therapist *t* ∈ *T*^*p*^ for each period *p* ∈ *P*. Moreover, the maximum number of patients that a therapist *t* has chosen to treat for the overall assignment is labeled as *a*_*t*_ ≥ 0, and ${{d}_{t}^{p}} \geq 0$ is a parameter that measures the therapist’s contribution to the assignment at a given period *p*. The latter allows to increase the number of active therapists at each period *p* instead of concentrating the assignment into a few.

Let $G = (\mathcal {C} \cup Q, E)$ be a bipartite graph, where *E* is the set of edges where edge {*ℓ*,*q*} represents the capability of the therapist group *q* ∈ *Q* to treat patients of category $\ell \in \mathcal {C}$.

**Fig. 3:**
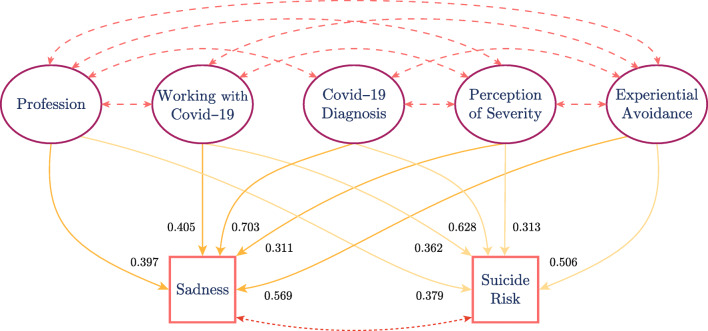
Relationships between variables with the WSLMV method. The weights are standardized

Let $c^{p}:E \to \mathbb {R}^{+}$ be a function over the set of edges representing the affinity between patients and therapists for period *p* ∈ *P*. This affinity function requires that, for any two therapists *t*_1_ and *t*_2_ with $q_{t_{1}} > q_{t_{2}}$, the relation $ {c}_{\ell ,t_{1}}^{p} > {c}_{\ell , t_{2}}^{p} $ holds for any category $\ell \in F_{q_{t_{1}}} \cap F_{q_{t_{2}}}$ and any period *p*. Here, the individual affinity between a therapist *t* ∈ *T*^*p*^ and a category $\ell \in \mathcal {C}$ is just ${c}_{\ell , q_{t}}^{p}$, and it is denoted as ${c}_{\ell ,t}^{p}$. This definition ensures that patients of category *ℓ* are more likely to be treated first by the therapists of the group that has more affinity with *ℓ* (Fig. 3).

Considering the practical requirements of the assignment described in Section 1, the following assumptions are taken in what follows: (a) Categories and groups do not evolve over periods. That is, an arriving patient belonging to a category cannot switch to another one on a future period. Likewise, therapists cannot switch groups. (b) The information provided for the assignment is limited to the number of patients of each category, and the group that each therapist belongs to. Also, as each therapist will contact their assigned patients to define a specific attention time in the corresponding period, the assignment does not need to take into account waiting and arrival times.

### A multi-periodic integer programming model

This formulation uses binary and integer variables. Let ${x}_{\ell ,t}^{p} \in \mathbb {Z}^{+}$ be the number of patients from category *ℓ* assigned to therapist *t* in period *p*, where $\ell \in \mathcal {C}$, *t* ∈ *T*^*p*^, and *p* ∈ *P*. Let ${y}_{t}^{p}$ be equal to one if therapist *t* treats at least one patient in period *p* and zero otherwise. Finally, ${z}_{\ell }^{p} \in \mathbb {Z}^{+}$ indicates the number of patients from category *ℓ* that cannot be assigned in period *p* due to a limited number of qualified therapists.

The *Multi-periodic Patients Assignation to Therapists Model* (*MPATM*) is stated as follows:

1 $$  \max  \underset{p \in P}{\sum} \underset{{(\ell,t)\in E^{p}}}{\sum} {c}_{\ell,t}^{p} {x}_{\ell,t}^{p} + \underset{p\in P}{\sum} \underset{{t \in T^{p}}}{\sum} {{d}_{t}^{p}} {{y}_{t}^{p}} $$

subject to

2 $$ \begin{array}{@{}rcl@{}} {\underset{r\in P}{\sum}} \underset{\ell\in \mathcal{C}}{\sum} {x}_{\ell,t}^{r} &\leq& a_{t} \quad\quad\quad\quad\forall t \in \underset{p\in P}{\bigcup} T^{p}, \end{array} $$

3 $$ \begin{array}{@{}rcl@{}} \underset{\ell \in \mathcal{C}}{\sum} {x}_{\ell,t}^{p} &\leq& a_{t} {{y}_{t}^{p}} \quad\quad \quad\forall p \in P,t \in T^{p}, \end{array} $$

4 $$ \begin{array}{@{}rcl@{}} {\sum}_{\ell \in \mathcal{C}} {x}_{\ell,t}^{p} &\geq& {{y}_{t}^{p}}  \quad\quad \quad\quad\forall p \in P,t \in T^{p}, \end{array} $$

5 $$ \begin{array}{@{}rcl@{}} \underset{{ t \in T^{0}}}{\sum} {x}_{\ell,t}^{0} + {z}_{\ell}^{0} & = & {b}_{\ell}^{0} \quad\quad\quad \quad  \forall \ell \in \mathcal{C}, \end{array} $$

6 $$ \begin{array}{@{}rcl@{}} \underset{{ t \in T^{p}}}{\sum} {x}_{\ell,t}^{p} + {z}_{\ell}^{p} & = & {b}_{\ell}^{p} + {z}_{\ell}^{p-1} \quad \forall p \in P\setminus \{0\}, \ell \in \mathcal{C}, \end{array} $$

7 $$ \begin{array}{@{}rcl@{}} |{T}_{q_{t}}^{p}| \left( \underset{{ \ell \in \mathcal{C}} }{\sum} {x}_{\ell,t}^{p} +1 \right) \geq \underset{\ell \in F_{q_{t}}}{\sum} \underset{q_{k} = q_{t}} {\underset{k\in T^{p}:}{\sum}} {x}_{\ell,k}^{p}  \forall p \in P,t \in T^{p}, \end{array} $$

8 $$ \begin{array}{@{}rcl@{}} &&x_{\ell,t}^{p} \in \mathbb{Z}^{+}  \quad\quad\quad \ \  \forall p \in P, (\ell,t) \in E^{p}, \end{array} $$

9 $$ \begin{array}{@{}rcl@{}} &&z_{\ell}^{p} \in \mathbb{Z}^{+}  \quad\quad\quad\quad   \forall p \in P, \ell \in \mathcal{C}, \end{array} $$

10 $$ \begin{array}{@{}rcl@{}} &&{y_{t}^{p}} \in \{0,1\}  \quad\quad\quad  \forall p \in P,t \in T^{p}. \end{array} $$

The objective function Eq. 1 aims to maximize the global affinity between patients and therapists and the latter’s total contribution to the assignment. Efficient solutions can be found selecting adequate values of *c* and *d*. In contrast, varying these parameters in an unbalanced fashion can result in one term of the objective dominating the other. When at least one bottleneck term is present, Punnen [66] discusses the optimization of the sum of the objectives for general combinatorial optimization problems.

Constraints Eq. 2 verify that the upper bound for the maximum number of patients assigned to each therapist, over all periods, is not exceeded. For each period, the sets of coupling constraints Eqs. 3 and 4 impose upper and lower bounds for the number of patients that each therapist can treat, and determine the active therapists who have at least one assigned patient. Without Eq. 3, there could be assignments for a therapist without capturing their contribution. Notice that Eq. 3 controls the assignment on a periodic basis, whereas Eq. 2 applies for the overall assignment. Constraints Eqs. 5 and 6 identify, for each category $\ell \in \mathcal {C}$, the number of assigned patients to any therapist and a possible number of patients who cannot be treated due to the limited number of qualified therapists in a specific period. Note that the number of patients to be assigned on a period *p* ∈ *P* ∖{0} includes the remaining patients from period *p* − 1. Finally, constraints Eq. 7 guarantee that the workload in every period is homogeneously distributed in each group of therapists. Here, for a given therapist *t* of group *q*_*t*_, the number of patients assigned to such a group divided by the number of therapists in *q*_*t*_ minus one is a lower bound for the number of patients that can be assigned to *t*.

Due to the effects of the Covid–19 emergency, there is not enough information to develop a reliable forecast to predict the demand for *MPATM*; thus, the information related to sets *S*^*p*^ and *T*^*p*^ is limited, knowing their actual values only on the current period. A solution strategy is proposed in the next section to tackle this limitation for the applicability of *MPATM*.

### Solution algorithms

As the goal of AEPPBE is to assign each patient to a therapist as soon as possible, without overloading each volunteer, it was suggested to break down *MPATM* in *ρ* independent assignments using the available information collected between periods *p* − 1 and *p* for each *p* ∈ *P* ∖{0}. This allows to simplify *MPATM* by dropping *p* from the variables and sets in *MPATM*. Thus, for a fixed period, the *Patients Assignation to Therapists Model* (*PATM*) is stated as follows:

11 $$  \max  \underset{{ (\ell,t)\in E }}{\sum} c_{\ell,t} x_{\ell,t} + \underset{{ t \in T }}{\sum} \widehat{d}_{t} y_{t} $$

subject to

12 $$ \begin{array}{@{}rcl@{}} \underset{{ \ell \in \mathcal{C} }}{\sum} x_{\ell,t} &\leq& \widehat{a}_{t} y_{t} \quad\quad \forall t \in T, \end{array} $$

13 $$ \begin{array}{@{}rcl@{}} \underset{{ \ell \in \mathcal{C} }}{\sum} x_{\ell,t} &\geq& y_{t} \quad\quad \ \ \ \forall t \in T, \end{array} $$

14 $$ \begin{array}{@{}rcl@{}} \underset{{ t \in T }}{\sum} x_{\ell,t} + z_{\ell} & =& \widehat{b}_{\ell} \quad\quad \ \ \ \forall \ell \in \mathcal{C}, \end{array} $$

15 $$ \begin{array}{@{}rcl@{}} |T_{q_{t}}| \left( \underset{{\ell \in \mathcal{C}}}{\sum} x_{\ell,t} +1 \right) &\geq& \underset{\ell \in F_{q_{t}}}{\sum} \underset{q_{k} = q_{t}} {\underset{k\in T}{\sum}} x_{\ell,k}\quad\forall t \in T, \end{array} $$

16 $$ \begin{array}{@{}rcl@{}} x_{\ell,t} &\in& \mathbb{Z}^{+}  \quad\quad \ \ \  \forall (\ell,t) \in E, \end{array} $$

17 $$ \begin{array}{@{}rcl@{}} z_{\ell} &\in& \mathbb{Z}^{+} \quad\quad  \ \ \  \forall \ell \in \mathcal{C}, \end{array} $$

18 $$ \begin{array}{@{}rcl@{}} y_{t} &\in& \{0,1\}  \quad \ \ \   \forall t\in T. \end{array} $$

The variables *x*_*ℓ*,*t*_ represent the number of patients from category *ℓ* assigned to therapist *t*, *y*_*t*_ take the value of one if therapist *t* treats at least one patient, and *z*_*ℓ*_ indicate the number of patients from category *ℓ* that cannot be assigned due to a limited number of qualified therapists.

Additionally, let *e*_*t*_ be the cumulative number of patients that have already been assigned to therapist *t* in previous periods. The remaining slots for *t* to treat patients during the current assignation is denoted as $\widehat {a}_{t}$, that is $ \widehat {a}_{t} = a_{t} - e_{t} $. To not overload volunteer therapists already treating some patients and give preference to those who do not have any assigned, the individual contribution of a therapist *t* for the current assignment $\widehat {d}_{t} $ is defined as

19 $$  \widehat{d}_{t} = \begin{cases} \max_{(\ell,\tau)\in E} c_{\ell, \tau} + q_{t} + a_{t} &\text{if } e_{t} = 0,\\ \max_{(\ell,\tau)\in E} c_{\ell, \tau} + q_{t} - \alpha e_{t} &\text{otherwise;} \end{cases} $$

where *α* ≥ 0 is such that $ \widehat {d}_{t} > 0$. This election also allows to balance the two terms that form the objective function and to first assign patients to the therapists of lower qualification that have not had a previous assignment. At last, let $\widehat {b}_{\ell }$ be the sum of the newly registered and remaining patients of category $\ell \in \mathcal {C}$ that could not be assigned on any previous periods.

Similarly, as in (*MPATM*), the objective function Eq. 11 aims to maximize the affinity between patients and therapists and the total contribution of therapists in the assignment. The sets Eqs. 12 and 13 impose upper and lower bounds for the number of patients that each therapist can treat, and determine the active therapists. For each category, constraints Eq. 14 identify the number of assigned patients to any therapist and possible remaining patients who cannot be treated due to the limited number of qualified therapists. Finally, constraints Eq. 15 guarantee that the workload is homogeneously distributed in each group of therapists.

Notice that constraints Eq. 14 in (*PATM*) allow unassigned patients; that is *z*_*ℓ*_ > 0 for some $\ell \in \mathcal {C}$. Here, the following necessary conditions must hold for any feasible assignment where there are no patients left to assign:

20 $$ \begin{array}{@{}rcl@{}} \sum\limits_{\ell \in \mathcal{C}} \widehat{b}_{\ell} &\leq& \sum\limits_{t\in T} \widehat{a}_{t}, \end{array} $$

21 $$ \begin{array}{@{}rcl@{}} \widehat{b}_{\ell} &\leq& \underset{\ell \in F_{q_{t}}}{\sum\limits_{t\in T:}} \widehat{a}_{t} \qquad \forall \ell \in \mathcal{C}. \end{array} $$

Here, inequality Eq. 20 guarantees that there are enough available appointments of each therapist to treat all patients, and inequalities Eq. 21 ensure treatment for each patient category.

A heuristic is presented to find feasible solutions of *MPATM*. Algorithm 1, uses model *PATM* at each period *p* ∈ *P* and iteratively updates $\hat a$ and $\hat d$. 
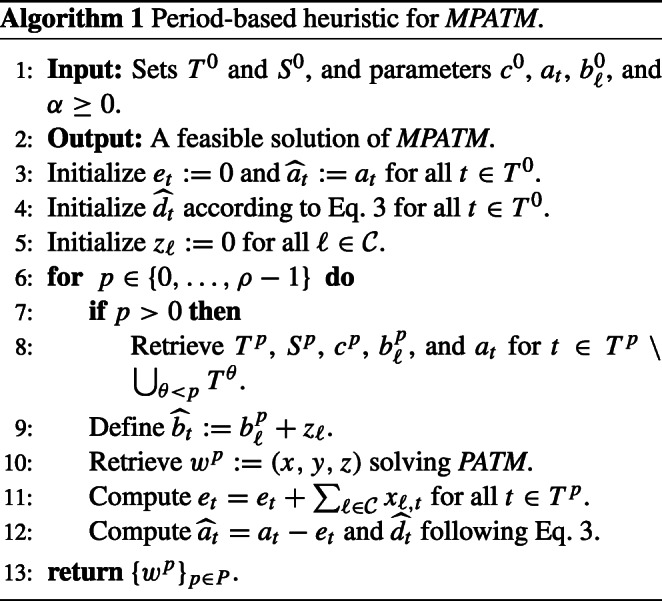


### Computational results

In this section, some computational experiments are presented. Initially, a set of instances with data from AEPPBE is solved using the Period-based heuristic. Then, simulated instances are constructed based on the real-world data to test the solution approach determined by Algorithm 1. This is tested against a priority-based strategy.

The integer programming models were solved to optimality using the optimization solver Gurobi 9.0.2 [33], in default settings, with its Python interface. All the experiments were performed in a matter of seconds on a MacBook Air 1.6 GHz Dual-Core Intel Core i5 with 8 GB RAM.

#### Real-world Instances

Results presented in this section were obtained from applying Algorithm 1 to real data provided by AEPPBE collected with the surveys introduced in Section 2.2.

An instance for *MPATM* is composed of (a) a set of periods, (b) a set of patients labeled by their categories, (c) a set of therapists, classified by their qualification, alongside the maximum number of patients they can treat, (d) the affinity between patients and therapists, and (e) the vector of individual contribution of each therapist in the overall assignment. Experimentation was performed over a set of three instances, labeled *I*_*i*_ for *i* ∈{1,…,3}, with different planning horizons. The first instance contains data from three periods, the second from two, and the last one has one period with accumulated data of patients before the development of this study.

For all instances, the set of volunteer therapists and the affinity values were fixed. Table 6 presents the number of patients per category for each instance. Moreover, in Table 7, information of the 63 participant therapists, classified by their qualification in 9 groups, is provided. The number of volunteers per group and the average, minimum, and maximum values for the upper bound on the number of patients that each group can treat are also included.

**Table 6:** Number of patients per category in each instance of (*PATM*)

Categories	Instances					
	*I*_1_			*I*_2_		*I*_3_
	0	1	2	0	1	0
0	–	–	–	–	–	–
1	1	1	–	–	–	9
2	–	–	1	1	1	4
3	7	12	10	7	15	93
4	–	1	–	–	1	2
5	1	1	1	–	–	3
6	3	4	1	2	1	20
7	1	31	2	8	3	47
8	3	6	4	1	14	81
9	5	7	5	–	3	35
Total	21	63	24	19	38	294

**Table 7:** Statistics associated with therapists

Group	Number of therapists	Average *a*	Min *a*	Max *a*
0	15	2.67	2	4
1	–	–	–	–
2	2	3.00	3	3
3	1	2.00	2	2
4	16	2.63	2	4
5	6	2.50	2	3
6	2	3.00	3	3
7	1	2.00	2	2
8	20	2.65	1	4

**Fig. 4:**
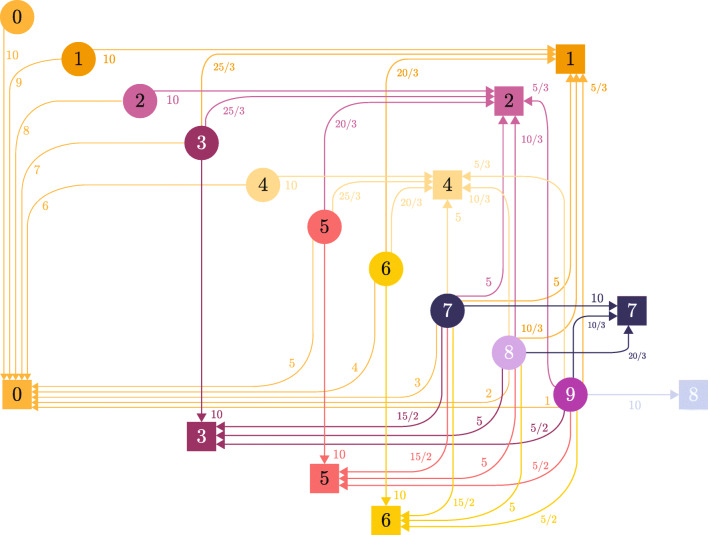
Affinity relationship between categories of patients and groups of therapists

Given a group of therapists *ν* ∈ *Q*, its affinity with the categories of patients $\mathcal {C}$ is defined by a decreasing numerical range according to the set *F*_*ν*_. Here, this range is normalized to guarantee that the affinity between categories and groups, with the best qualification to treat them, is the highest (see Figs. 1 and 2). The affinity relationship is captured in Fig. 4. Here, patient categories are represented by circles, and the groups of therapists are pictured with squares, each identified according to its label in $\mathcal {C}$ and *Q*, respectively. Each arc depicted in the graph connects a category of patients with a group of therapists, representing that such a group can treat the category. Thus, if there is no arc connecting a category with a group, it means that the group is not qualified to treat the category. This relationship implicitly determines *F*_*ν*_ for each *ν* ∈ *Q* and the function of affinity *c*. In the latter, *c*_*ℓ*,*t*_ corresponds to the affinity of category *ℓ* ∈ *C* with the group of therapists containing *t*. In order to exemplify this, in Fig. 4, group *ν* = 0 can treat any category of patients, and the affinity coefficients are *c*_*ℓ*,*t*_ = 10 − *ℓ*, for all *t* such that *q*_*t*_ = 0. To compute an affinity function for the other groups, let us enumerate the elements of *F*_*ν*_ by a set *J* respecting the natural ordering by category and starting at zero. Let us denote *ι*_*ℓ*_ the index of Category *ℓ* given by *J*. Then the affinity value for all therapists *t* of Group *ν* = *q*_*t*_ is given by

$$ c_{\ell, t} = |F_{0}| - \iota_{\ell} \frac{|F_{0}|}{|F_{\nu}|} $$

for all *ι*_*ℓ*_ ∈ *J*. The resulting affinity function is consistent with the definition from Section 4.1. To illustrate this, consider *F*_3_ = {3,7,8,9} along the ordered set *J* = {0,1,2,3}, then Category 3 is indexed by *ι*_3_ = 0, Category 7 is indexed by *ι*_7_ = 1, and so on. Then the affinity value between Category 7 and any therapist *t* in Group 3 is $ c_{7,t} = \frac {3}{4} |F_{0}| = \frac {15}{2}$ and clearly $ c_{7,t} > c_{7,t_{1}} $ for any *t*_1_ in Group 0. At last, the individual contribution of each therapist *t* ∈ *T* to the overall assignment follows from Eq. 19 using *α* = 2.

In instances *I*_1_ and *I*_2_, all the requirements imposed for patient treatment were satisfied, and every patient was assigned. Instance *I*_3_ also followed the requirements, yet some patients were left without treatment, as there were not enough qualified therapists to cover such massive demand. This can be expected from Tables 6 and 7, where the number of qualified therapists to treat Category 3 is scarce in contrast to the 93 registered patients. Despite this result, it is important to point out that instance *I*_3_ was collected only for experimentation purposes as it had data of previously treated patients.

**Fig. 5:**
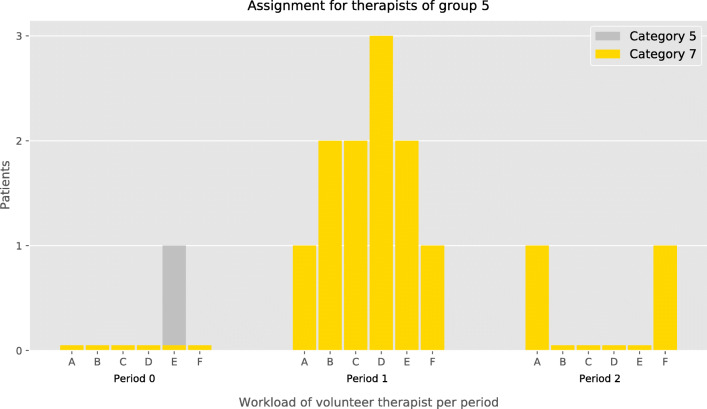
Periodic assignment of fifth group for instance *I*_1_. Therapists are labeled with capital letters *A* to *F*

In particular, results for instance *I*_1_ are summarized and presented in Table 8. There, for each period and category, the number of patients assigned to each group of therapists is reported. For instance, Category 3 has 29 patients for the overall planning horizon. Of these, in period 0, four were assigned to volunteer therapists of Group 0, two to Group 2, and one to Group 3, respectively. Moreover, a detailed assignment for Group 5 is presented in Fig. 5. This group had six volunteer therapists labeled with capital letters from *A* to *F*. Each one was qualified to treat Covid–19 confirmed patients or anyone with any level of Emotional Risk (i.e., Categories 5,7,8, and 9). At last, a high level of participation from therapists was observed. That is, if the number of patients assigned to a group is greater or equal than the number of available therapists, then at least one patient is assigned to each one. For example, in period 0, five patients of Categories 1 and 3 were assigned each to five different therapists of Group 0.

**Table 8:** Number of patients per category assigned to each group of therapists for (*PATM*) in instance *I*_1_

Categories	Groups of volunteer therapists																										
	Period 0									Period 1									Period 2								
	0	1	2	3	4	5	6	7	8	0	1	2	3	4	5	6	7	8	0	1	2	3	4	5	6	7	8
0	–	–	–	–	–	–	–	–	–	–	–	–	–	–	–	–	–	–	–	–	–	–	–	–	–	–	–
1	1	–	–	–	–	–	–	–	–	1	–	–	–	–	–	–	–	–	–	–	–	–	–	–	–	–	–
2	–	–	–	–	–	–	–	–	–	–	–	–	–	–	–	–	–	–	–	–	1	–	–	–	–	–	–
3	4	–	2	1	–	–	–	–	–	12	–	–	–	–	–	–	–	–	9	–	1	–	–	–	–	–	–
4	–	–	–	–	–	–	–	–	–	–	–	–	–	1	–	–	–	–	–	–	–	–	–	–	–	–	–
5	–	–	–	–	–	1	–	–	–	–	–	–	–	1	–	–	–	–	–	–	–	–	1	–	–	–	–
6	–	–	–	–	1	–	2	–	–	–	–	–	–	–	–	4	–	–	–	–	–	–	1	–	–	–	–
7	–	–	–	–	–	–	–	1	–	2	–	2	1	14	11	–	1	–	–	–	–	–	–	2	–	–	–
8	–	–	–	–	–	–	–	–	3	–	–	–	–	–	–	–	–	6	–	–	–	–	–	–	–	–	4
9	–	–	–	–	–	–	–	–	5	–	–	–	–	–	–	–	–	7	–	–	–	–	–	–	–	–	5
Total	5	0	2	1	1	1	2	1	8	15	0	2	1	16	11	4	1	13	9	0	2	0	2	2	0	0	9

#### Simulated instances

To study the behavior of the problem and the chosen value of *c* for several periods, random instances were generated based on the real-world data. Six instances were studied using a different number of periods *ρ*.

Fixing a period *p* ∈{0,…,*ρ* − 1}, the set of patients *S*^*p*^ was sampled as follows: The number of patients present at Category *ℓ* was sampled from a Poisson distribution with mean *λ*_*ℓ*_. The value of *λ*_*ℓ*_ was taken as the average of the mean number of patients at Category *ℓ* from instances *I*_1_ and *I*_2_ reported in Table 6. As Category 0 did not present any recorded patient, the value *λ*_0_ = *ρ*^− 1^ was selected to expect at least one patient.

In contrast, the set of therapists *T*^*p*^ was simulated as a random process starting from the set *T*^0^. This set was chosen such that the number of therapists from each group was the same as in Table 7. Then, for periods *p* ≥ 1, therapists were added to each group at rate $\lambda = \frac {3}{4}$ and removed at rate $\mu = \frac {1}{8} $, both following a Poisson distribution. The maximum number of patients that each therapist in $T = \bigcup _{p} T^{p}$ was able to treat was sampled from a uniform distribution with its lower bound matching the fourth column of Table 7, and its upper bound was modified accordingly for each instance such that the total number of patients would be less than the total number of therapists times this value. Notice that this does not guarantee treatment for all patients. This is explained by the fact that the set *T*^*p*^ evolves after every period, thus it can remove some therapists that still had available appointments after the assignment at period *p* − 1 or not generate enough therapists of a certain required group. Moreover, the set of inequalities Eq. ?? is not necessarily satisfied.

In this patient assignment problem, a second policy might also perform well. Thus, one can think of a category-based extension of Algorithm 1 satisfying that every therapist treats an homogeneous number of patients of each category. Here, at each period, a solution of *PATM* is found for each category of patients sequentially and following the natural order of $\mathcal {C}$. This coincides with the prioritization order for treating psychological risk; i.e., an attempt to find an assignment for patients of Category 0 is done first, then another for patients of Category 1, and so on. Algorithm 2 describes this alternative policy. 
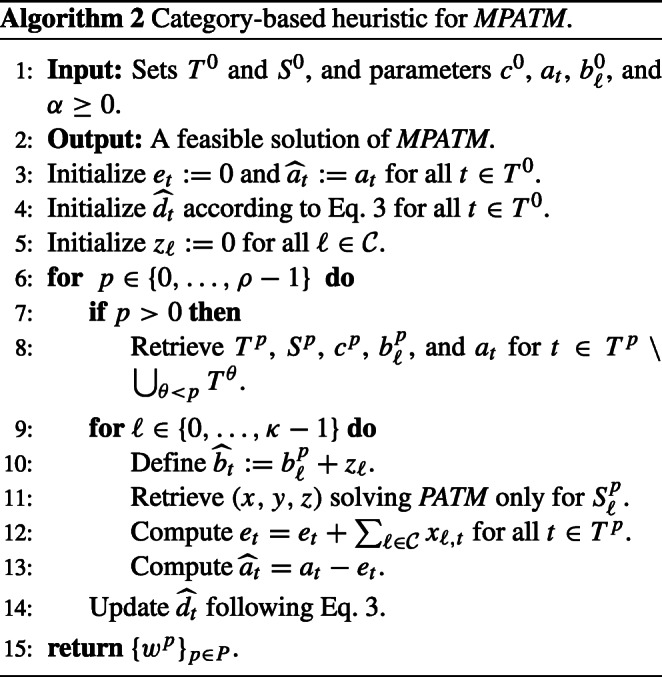


A summary of the instances and the corresponding results for applying Algorithms 1 and 2 is displayed in Table 9. There, the first four columns present the number of periods *ρ*, the number of patients and therapists for the overall assignment, and the maximum number of patients that any therapist can treat. Next, for each algorithm, the cumulative objective value and the first untreated category, alongside the number of untreated patients from such category, are given.

**Table 9:** Results of simulated instances

Periods *ρ*	Number of patients	Number of therapists	Max *a*	Algorithm 1		Algorithm 2	
				Objective value	1st Untreated Category	Objective value	1st Untreated Category
5	184	90	3	3 856	–	4 096	–
7	234	108	3	4 852	3 (5)	5 157	3 (7)
10	334	116	4	6 935	–	7 266	3 (10)
12	394	134	4	8 266	–	8 661	3 (2)
15	525	144	6	10 325	–	11 443	–
60	1 947	465	7	36 502	–	40 5098	3 (14)

From the results, the cumulative objective value of the policy followed by Algorithm 1 is lower than the value reported for Algorithm 2. This is explained since the policy adopted in the latter does not satisfy the periodic homogeneity constraint of workload, which is a clear drawback for the main purpose of *MPATM* and its real-world application. In addition, Algorithm 1 presents no untreated patients in most of the studied instances, whereas the contrary happens for Algorithm 2. For the only case where there were untreated patients in the Period-based heuristic, their number was lower than the one reported from the Category-based heuristic.

It is important to notice that in both results, the reported category with untreated patients is Category 3. Considering the data from Table 6, the value $\lambda _{3} = \frac {31}{3}\approx 10 $ is relatively high for the number of therapists belonging to Group 3 in *T*^0^. Thus, even though the number of therapists from Group 0 is high, after some periods most workload from Categories 0 to 3 will be assigned to these therapists.

## Discussion

This research aimed to provide an effective assignation model from patients to therapists depending on psychological criteria. Statistical methods were used to analyze the influence of psychological variables on mental health impact due to Covid–19 in the Ecuadorian population. Later, patients and therapists were divided into categories and groups to build an integer programming model.

While there remains much ground to be covered in the management of mental health impact of Covid–19, it is valuable to note that an integer programming model could be an useful approach. The present work shows that applied science can provide tools to address the impact of Covid–19 with reduced distress and effective administration of psychological treatment in the Ecuadorian context. Mathematical modelling can offer a helpful tool for understanding and modeling health systems.

In the beginning, during weekly supervision meetings, members of AEPPBE expressed their concern regarding patients’ needs, lack of specific competences, or being unfamiliar with Telebehavioral Health. Later, symptoms of perceived distress were observed in the volunteers. This distress involved anxiety symptoms, a feeling of overwhelming around volunteering, and a feeling of being unable to cope with certain categories of patients. Also, volunteers had an uncertain assignation of workload per day, generating high levels of tension. This agrees with Carleton [18], Anderson et al. [4] and Lake and LaBar [41], as these kinds of assignments could lead to anxiety depending on the uncertainty of the weekly number of patients and their needs.

Previous crisis events, such an earthquakes in Ecuador, showed intervention from NGOs mostly focused on psychosocial support [38, 78]. Nevertheless, none of the previous volunteer work has faced the challenges of the Covid–19 pandemic. Moreover, one of the problems related to Covid–19 was healthcare system deficits, both in material and human resources [37].

It is important to remark that the main contribution of this work is to solve a real-world application successfully. The integer programming model (*PATM*) provided a solution to the problems mentioned above. First, a proper assignment of patients provided more certainty to the volunteers. Grupe and Nitschke [31] described the link between uncertainty and anxiety; thus, applying the assignation model supports therapist self-care, which Norcross and Phillips [57] stated as an essential practice during the Covid–19 pandemic. Additionally, it helped to accurately assign each therapist according to their capabilities and preferences. Besides, locally, this assignation helped AEPPBE to align with occupational objectives regarding the well-being of volunteers following the guidelines of Ministerio de Salud Pública de Ecuador [51]. Furthermore, computing and processing time took less than a minute per period, which contrasts with the reported time to compute the empirical assignment. Before (*PATM*) was introduced, this task was done by two members of AEPPBE, spending around three days to find a feasible solution per period. Summarizing, (*PATM*) contributed with a technical tool to distribute scarce human resources needed during the pandemic, being the result of collaborative work between NGOs and applied science.

Furthermore, this model could be applied to address problems related to effective assignation in healthcare systems as it is not limited to the Covid–19 emergency. For instance, it can be used during other emergencies and crisis events (e.g., earthquakes, volcanic eruptions, floods, or new pandemics) within different populations and with other psychological variables. Thus, the proposed optimization techniques can be used to enhance mental health care in the community, not only by psychologists but also by crisis and psychological first aid supporters. Besides, the blindness and equability, regarding how patient’s data is processed in the model, could aid in reducing bias in experimental Psychotherapy research.

As with any modelling study, there are limitations to discuss. First, cross-sectional sampling was used, limiting predictions on future data. Second, the urgent need for assessment during the pandemic required the use of mono-items to evaluate certain psychological variables. This further limited the applicability of this study to people with internet accessibility only. Third, the information provided by volunteers cannot be certified as there are prior legal limitations related to the regulation of psychological practices in Ecuador. Similarly, web surveying did not guarantee that patients were personally filling their own assessment. Finally, the limited availability of data during the emergency imposed the formulation of a heuristic to approximately solve the multi-periodic model.

## Appendix

**Table 10:** Summarised notation for Section 4

Symbol	Definition	Description
*P*	Set of periods	Finite set
*ρ*	Number of periods	*ρ* = |*P*|
$\mathcal {C}$	Set of categories of patients	Finite set
*κ*	Number of categories of patients	$\kappa = |\mathcal {C}|$
*S*^*p*^	Set of patients to be assigned at period *p*	Finite set
$S_{\ell }^{p}$	Set of patients of category *ℓ* to be assigned at period *p*	Finite set
$b_{\ell }^{p}$	Number of patients of category *ℓ* to be assigned at period *p*	${b}_{\ell }^{p} = |{S}_{\ell }^{p}|$
*Q*	Set of groups of therapists	Finite set
*σ*	Number of groups of therapists	*σ* = |*Q*|
*T*^*p*^	Set of volunteer therapists available at period *p*	Finite set
$T_{\nu }^{p}$	Set of therapists of group *ν* available at period *p*	Finite set
*a*_*t*_	Maximum number of patients that therapist *t* has chosen to treat	$t \in \bigcup _{p} T^{p}, a_{t} \geq 0$
	in the overall assignment	
*q*_*t*_	Qualification / group of therapist *t*	*q*_*t*_ ∈ *Q*,*t* ∈ *T*^*p*^, *p* ∈ *P*
$F_{q_{t}}$	Set of categories of patients that a therapist of rank *q*_*t*_ can treat	$F_{q_{t}} \subseteq \mathcal {C}$
*G*	Bipartite graph $G=(\mathcal {C} \cup Q, E)$	Undirected graph
*E*	Set of edges where edge {*ℓ*,*q*} is the capability of group *q* ∈ *Q*	Finite set
	to treat patients of category $\ell \in \mathcal {C}$	
*c*^*p*^	Function for the affinity between patients and therapists for period *p*	$c^{p}: E \to \mathbb {R}^{+}, p \in P$
${d_{t}^{p}}$	Therapist *t* contribution to the assignment at period *p*	Non-negative value
$x_{\ell ,t}^{p}$	Number of patients from category *ℓ* assigned to therapist *t* at period *p*	Integer decision variable
${y_{t}^{p}}$	Variable taking the value of one if therapist *t* treats any patient at period *p*	Binary decision variable
$z_{\ell }^{p}$	Number of patients from category *ℓ* that cannot be assigned at period *p*	Integer decision variable
*x*_*ℓ*,*t*_	Number of patients from category *ℓ* assigned to therapist *t*	Integer decision variable
*y*_*t*_	Variable taking the value of one if therapist *t* treats any patient	Binary decision variable
*z*_*ℓ*_	Number of patients from category *ℓ* that cannot be assigned	Integer variable
*e*_*t*_	Cumulative number of patients that have already been assigned to	Integer value
	therapist *t* in previous periods	
$\hat {a}_{t}$	Remaining slots for therapist *t* to treat any patients	$\hat {a}_{t} = a_{t} - e_{t}$
$\hat {d_{t}}$	Contribution of therapist *t* for the assignment	Non-negative value
$\hat {b}_{\ell }$	Sum of newly registered and remaining patients of category *ℓ* that	Integer value
	could not be assigned on any previous period	
